# Spanish modified version of the palliative care outcome scale–symptoms renal: cross-cultural adaptation and validation

**DOI:** 10.1186/s12882-016-0402-8

**Published:** 2016-11-18

**Authors:** Daniel Gutiérrez-Sánchez, Juan P. Leiva-Santos, Rosa Sánchez-Hernández, Domingo Hernández-Marrero, Antonio I. Cuesta-Vargas

**Affiliations:** 1 Fundación Cudeca, Av. del Cosmos, s/n, 29631 Arroyo de la Miel, Málaga, Spain; 2 Cuidados Paliativos. Hospital de Manacor, Carretera de Manacor-Alcudia, Mallorca, Islas Baleares, Spain; 3 Departamento de Nefrología, Hospital General de Villalba, Carretera de Alpedrete a Moralzarzal M-608 Km 41, 28400 Collado Villalba, Madrid, Spain; 4 Departamento de Nefrología, Hospital Regional Universitario de Málaga, Av Carlos Haya s/n, 29010 Málaga, Spain; 5 Instituto de Investigación Biomédico de Málaga (IBIMA), Málaga, Spain; 6 Departamento de Fisioterapia, Universidad de Málaga, C/ Arquitecto Francisco Peñalosa, Ampliación Campus Teatinos, 29071 IBIMA, Málaga, Spain; 7 School of Clinical Sciences, Faculty of Health at the Queensland University of Technology, Queensland, Australia

**Keywords:** Outcome measure, Symptoms, ACKD, Psychometrics

## Abstract

**Background:**

Patients with chronic kidney disease (CKD) have a high symptoms burden that is related to a poor health-related quality of life (HRQoL) and high costs of care. Validated instruments may be useful for assessing the symptoms and monitoring outcomes in these patients. The Palliative care Outcome Scale-Symptoms Renal (POS-S Renal) is a patient-reported outcome measure for assessing symptoms in CKD stage 4–5. This study is the first cross-cultural adaptation and psychometric analysis of this clinical tool. The purpose of this study is to carry out a cross-cultural adaptation of the POS-S Renal for Spanish-speaking patients, and to perform an analysis of the psychometric properties of this questionnaire.

**Methods:**

The English version of the POS-S Renal was culturally adapted and translated into Spanish using a double forward and backward method. An expert panel evaluated the content validity. The questionnaire was pilot-tested in 30 patients. A total of 200 patients with CKD stage 4–5 filled in a modified Spanish version of the POS-S Renal and the MSAS-SF. Statistical analysis to evaluate the psychometric properties of the questionnaire was carried out.

**Results:**

The content validity index (CVI) was 0.97, which indicated that the content of the instrument is an adequate reflection of the symptoms in advanced CKD (ACKD). The factor analysis indicated a two-factor solution explaining 35.05% of total variance. The confirmatory factor analysis (CFA) demonstrated that the two factor model was well supported (comparative fit index = 0.98, root mean square error of approximation = 0.068). This assessment tool demonstrated a satisfactory test–retest reliability (r = 0.909 to factor 1, r = 0.695 to factor 2, r = 0.887 to total score), good internal consistency to factor 1 (α = 0.78) and moderate internal consistency to factor 2 (α = 0.56). Concurrent criterion-related validity with MSAS-SF was also demonstrated, with r = 0.860, which indicated a high degree of correlation with a validated instrument that has been used in patients with ACKD.

**Conclusions:**

The Spanish modified version of the POS-S Renal is a reliable and valid instrument that can be used to assess symptoms in Spanish patients with CKD stage 4–5.

**Electronic supplementary material:**

The online version of this article (doi:10.1186/s12882-016-0402-8) contains supplementary material, which is available to authorized users.

## Background

A patient with Advanced Chronic Kidney Disease (ACKD)(defined, according to the international guidelines, as chronic kidney disease at stage 4 or 5 with a glomerular filtration rate < 30 ml/min) has an increased burden of physical and psychological symptoms that are associated with a poor health-related quality of life (HRQoL) [1–3]. Symptoms such as tiredness, pruritus, constipation, pain, sleep alterations, anxiety, dyspnoea, nausea, restless legs and depression have a high prevalence in these patients [4, 5]. As uncontrolled symptoms at the end of life lead to greater suffering, their treatment in the advanced phases of the disease is a priority [6].

Patient reported outcome (PRO) instruments are generally used to assess patients’ functional status, quality of life and symptoms [7]. This is especially important in patients with chronic diseases such as chronic kidney disease (CKD), in which PRO measures can be used to evaluate, monitor, and facilitate the introduction of interventions [8].

The use of validated scales may therefore be useful for standardising the evaluation of symptoms and monitoring the healthcare outcomes of these patients [9]. Because of the variety of symptoms that might be suffered by a kidney patient, tools that can evaluate a wide range of symptoms are recommended. In addition, it is useful to have instruments that can be administered pre- and post-dialysis start, in order to better monitor patients as they progress through CKD stages. Among the tools most used for the study of symptoms in kidney patients are questionnaires that are not specific to renal patients, such as the Memorial Symptom Assessment Scale-Short Form(MSAS-SF) [10], questionnaires modified for use with patients on dialysis, such as the Edmonton Symptom Assessment System (ESAS) [11], and other specific questionnaires that have been developed to measure symptoms in ACKD, such as the Dialysis Symptom Index (DSI) [12] and the Palliative care Outcome Scale-Symptoms Renal (POS-S Renal) [13].

There are no specific symptom assessment questionnaires for CKD patients that have been adapted to the Spanish culture, Spanish being one of the most commonly spoken languages in the world and that enable us to evaluate the intensity of the various symptoms in this population [14]. The POS-S Renal is a questionnaire that has been designed to evaluate symptoms in ACKD, and it has demonstrated its usefulness in clinical practice and in research [13, 15]. Thus it is a tool recommended for the evaluation of symptoms in this population [9, 16, 17]. There are at present no studies that have assessed the psychometric properties of this questionnaire.

The aims of this study were to carry out a cross-cultural adaptation of the POS-S Renal into Spanish and to perform an analysis of test-retest reliability, internal consistency, internal structure, and concurrent criterion-related validity with the MSAS-SF, a previously validated PRO measure which has been used in patients with ACKD [18–20].

## Methods

This study was conducted in two principal phases: translation and cross-cultural adaptation, and then validation.

### Phase 1: Translation and cross-cultural adaptation

#### Translation

Translation of the original English version of the POS-S Renal was performed using a double forward and backward method following recommended guidelines [21]. This method includes: 1) two forward translations from the source language into the target language; 2) reconciliation of the two versions; 3) two backward translations from the target language version to the source language; and 4) reconciliation of the two versions and comparison with the original to create a consensus document.

**Fig. 1:**
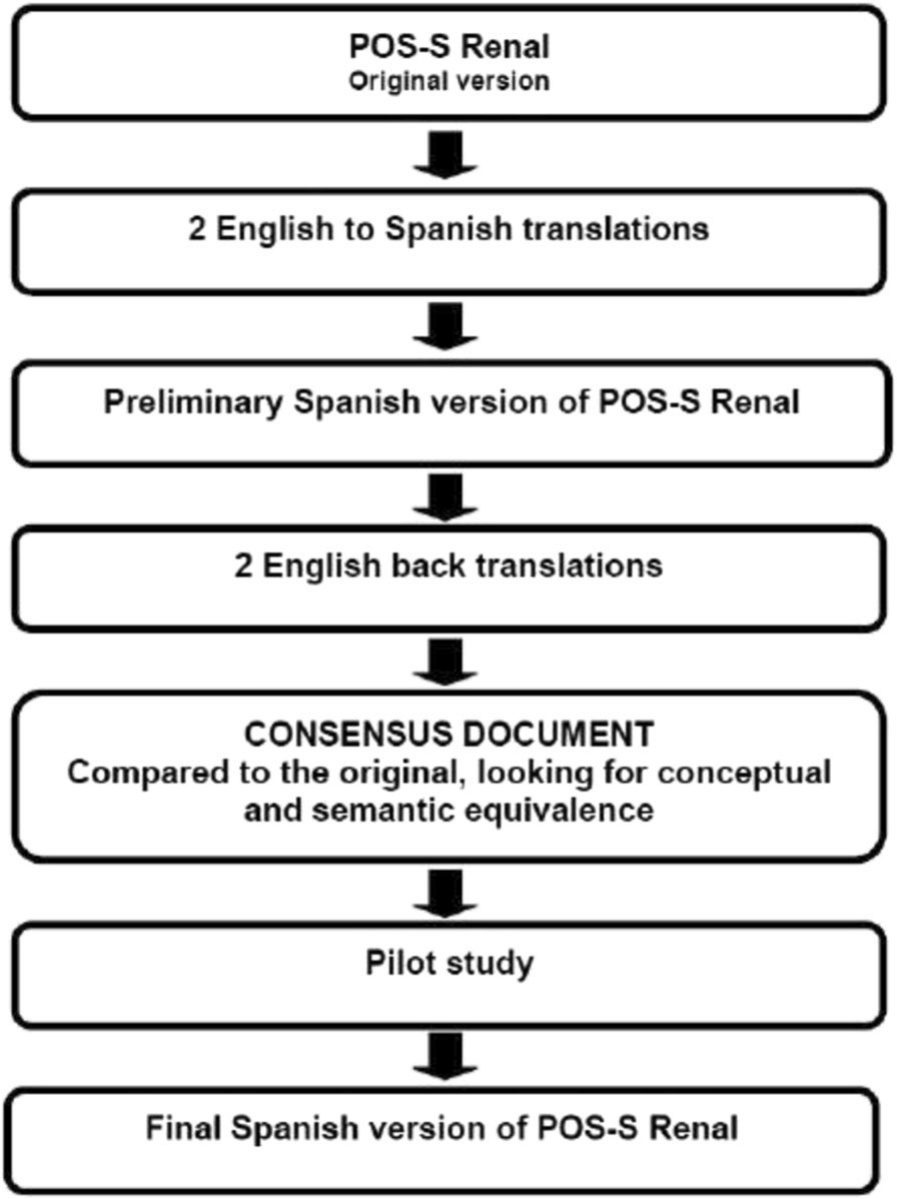
Flowchart of the translation of the POS-S Renal from English to Spanish. Two forward translations were performed by two translators. Two native English translators performed a backward translation. The back-translated version was compared with the original version to create a consensus document, which was pilot tested to provide a final version

There were two independent translations into Spanish. The two versions were compared and, after discussion, merged into a single document: the preliminary Spanish version of POS-S Renal. Two native English translators performed a backward translation of this version to the source language to make, after discussion, a single document. This document was compared with the original to check the conceptual and semantic equivalence between the two texts (Fig. 1).

#### Content validity

Content validity occurred during this phase to evaluate the degree to which the content of the instrument is an adequate reflection of the construct to be measured. An expert panel review was conducted to assess content validity. A total of 14 healthcare professionals (experts in palliative care and nephrology), researchers, patients with ACKD and carers of ACKD patients were invited to join the expert panel, but only eight of them (six healthcare professionals, one patient and one carer) agreed to participate. The other six declined because of lack of time (*n* = 4) or lack of knowledge about the subject (*n* = 2). Content validity was determined using the COSMIN checklist [22]. Individually, the expert participants were asked to rate their level of agreement with the relevance of each item. One symptom (cramps) was added after discussion. The response choices for this item were set to be identical to the response choices for the other 17items. This symptom was prevalent (29%), but had not previously been included in this symptom assessment tool. Content validity was determined using the Lawshe content validity index (CVI);employing the formula suggested by Lawshe, the CVI was 0.97, with items ranging from 0.75 to 1.00, which is acceptable, according to Lawshe and Davis [23, 24].

#### Pilot study

A pilot study was conducted with 30 patients to identify problematic items and to ensure that the adapted questionnaire was understandable and acceptable to the patients.

### Phase 2: Validation study

#### Sample, setting and data collection

Two hundred (200) patients (aged 66.45 ± 14.52, 65% male) participated in the present study. Their socio-demographic and clinical characteristics are shown in Table 1. All participants were recruited at the Nephrology Service at Carlos Haya University Hospital in Málaga (Spain), and, after receiving information about the project, signed to give their informed consent. The inclusion criteria were: adult Spanish-speaking patients with ACKD receiving dialysis or renal conservative management (without dialysis).

**Table 1:** Socio-demographic and Clinical Characteristics of the Sample (*N* = 200)

Characteristics	Tota (*N* = 200)	Conservative management group (*n* = 139)	Dialysis group (*n* = 61)
Age (mean, SD)	66.45 (±14.5)	69.65 (±12.8)	59.15 (±15.4)
Gender			
Male	130 (65%)	95 (68%)	35 (57.4%)
Female	70 (35%)	44 (32%)	26 (42.6%)
Ethnicity			
Caucasian	199 (99.5%)	138 (99.3%)	61 (100%)
Spanish descent	193 (96.5%)	132 (94.9%)	61 (100%)
British descent	3 (1.5%)	3(2.2%)	0 (0%)
German descent	3 (1.5%)	3(2.2%)	0 (0%)
Indian	1 (0.5%)	1(0.7%)	0 (0%)
Marital status			
Married	134 (67%)	94 (68%)	40 (65.6%)
Not married	66 (33%)	45 (32%)	21 (34.4%)
Causes of CKD			
Renal vascular disease	66 (33%)	56 (40.5%)	10 (16.4%)
Diabetic nephropathy	32 (16%)	22 (16%)	10 (16.4%)
Primary glomerular disease	17 (8.5%)	9 (6.5%)	8 (13.1%)
Polycystic kidneys	13 (6.5%)	8 (6%)	5 (8.2%)
Unknown aetiology	27 (13.5%)	19 (14%)	7 (11.5%)
Others	45 (22.5%)	24 (17%)	21 (34.4%)
Barthel index (mean, SD)	94.8 (±9.8)	94.93 (±9.5)	94.59 (10.3%)
Charlson comorbidity index			
2–3	83 (41.5%)	56 (40.3%)	27 (44.3%)
4–5	85 (42.5%)	58 (41.7%)	27 (44.3%)
≤ 6	32 (16%)	25 (18%)	7 (11.4%)

By contrast, there were three exclusion criteria: the patient’s refusal to participate in the study, mild cognitive impairment and the patient being under the age of 18 years. The data were collected between April 2015 and September 2015.

Data were collected during face-to-face interviews. When possible, the questionnaires (POS-S Renal and MSAS-SF) were self-completed. Otherwise, the interviewer read out and filled in the instruments according to the patient’s responses. In the case of patients managed conservatively (without dialysis), the interviews were conducted in the routine medical consultation room, whereas patients in dialysis were interviewed in the dialysis room. The interviewers first recorded the patient’s clinical socio-demographic data, and afterwards asked the patients to complete the POS-S Renal and the MSAS-SF.

### Questionnaires

#### POS-S renal

This is a patient-completed questionnaire that has been developed to assess symptoms in ACKD. The questionnaire identifies the presence and severity of 17 symptoms during the week prior to the completion of the questionnaire [13]. If the symptom is present, the intensity of each symptom is rated on a 5-point Likert scale (0 to 4) ranging from ‘none’ to ‘overwhelming’.

In addition, the questionnaire provides open fields to list the main problems experienced. This open-ended question gives the patient the opportunity to emphasise the importance of a symptom, or to indicate other symptoms not included in the questionnaire. In addition, a symptom severity score can be calculated from the questionnaire as a whole. If one apportions a number to the range of severity of each symptom (from 0 to 4), the maximum global symptom severity score is 68. This simple tool is used widely and is recommended as the tool of choice for ACKD [9]. The original questionnaire is available from: http://pos-pal.org/maix/pos-s-in-english.php.

#### MSAS-SF

The MSAS-SF is a validated self-reporting questionnaire that includes 32 highly common physical and psychological symptoms [10]. It assesses the presence and severity of 28 physical symptoms on a 5-point Likert scale (from 0 = ‘not bothersome’ to 4 = ‘very bothersome’) and the presence and frequency of four psychological symptoms on a 4-point Likert scale (from 1 = ‘rarely present’ to 4 = ‘almost constantly present’).

The MSAS-SF subscales include: 1) the Global Distress Index (GDI), measuring four psychological symptoms and six physical symptoms; 2) the Physical Symptom Distress Score(PHYS) comprising 12 common physical symptoms; and 3) the Psychological Symptom Distress Score (PSYCH), including six common psychological symptoms. The total number of symptoms (TNS) is the sum of symptoms experienced. The scoring procedure follows the instructions of Portenoy et al. for the short form [25]. The MSAS-SF has been cross-culturally adapted to Spanish speakers and has shown adequate psychometric properties [26]. It has been widely used in patients with ACKD [18–20].

#### Psychometric evaluation of the questionnaire

##### Reliability

Reliability was considered as test-retest reliability. To evaluate this, 73 patients completed the Spanish POS-S Renal twice, separated by 7 days. Pearson’s r correlation coefficient was used for assessment of the test-retest reliability.

##### Internal consistency

Internal consistency concerns whether there is a satisfactory degree of interrelatedness among the items. To evaluate this psychometric property, Cronbach’s α coefficients were calculated using the intraclass correlation coefficient type 2.1two-way mixed effects model, where people effects are random and measures effects are fixed (ICC_2.1_) [27].

##### Internal structure

Internal structure refers to the degree to which the scores for a questionnaire are an adequate reflection of the dimensionality of the construct to be measured. Exploratory factor analysis (EFA) with maximum likelihood extraction (MLE) and varimax rotation was used to evaluate this psychometric property. To confirm the factor model, we performed confirmatory factor analysis (CFA).

##### Concurrent criterion-related validity

Concurrent criterion-related validity refers to the degree to which the scores for an instrument are associated with scores for other instruments that are intended to evaluate similar constructs. This psychometric property was measured using the MSAS-SF as a reference, and Pearson’s r correlation coefficient was used to examine correlations between factor 1 of POS-S Renal and MSAS-SF total score, between factor 2 of POS-S Renal and MSAS-SF total score, and between the total score of POS-S Renal and MSAS-SF.

### Statistical analysis

Descriptive statistical analysis was used to determine the means and standard deviation of the sociodemographic and clinical variables and the POS-S Renal. We tested the differences in the responses from patients in dialysis and patients in conservative management using one-way analysis of variance (ANOVA).

Statistical analysis was conducted to assess the test-retest reliability, internal consistency, internal structure and concurrent criterion validity. The sample distribution was determined by Kolmogorov–Smirnov (KS) test. The Pearson’s r correlation coefficient used the criteria of: poor (an r < 0.49), fair (0.50 ≤ r ≤ 0.74), and strong (an r > 0.75) [28]. We assessed the measurement model of the POS-S Renal using CFA. The model fit indices included chi-square (*χ* ^2^), root mean square error of approximation (RMSEA), and the comparative fit index (CFI). For RMSEA, values of 0.08 or below indicate a close fit [29]. This analysis was conducted using SPSS version 20 and LISREL 8.80 [30].

## Results

### Translation and cultural adaptation

The POS-S Renal was translated and back-translated without language difficulties to provide the Spanish modified version of the instrument (Additional file 1). The mean ± SD time to complete the questionnaire at the first administration was 7 ± 2.5 min. The Spanish modified version of the POS-S Renal showed a low proportion of missing responses (this did not exceed 2%). The characteristics of the study sample are shown in Table 1.

Response from patients in dialysis and patients having conservative management showed no significant differences (*p* = 0.130 to factor 1 and *p* = 0.455 to factor 2).

### Pilot study

The translated and modified version of the POS-S Renal proved to be comprehensible and easy to complete during the pilot testing, and changes in format were not needed. The questionnaire was readable and acceptable by the target population (Additional file 2).

### Validation study

#### Test-retest reliability

The test–retest reliability was satisfactory. Pearson’s correlation coefficient was: r = 0.909 to factor 1, r = 0.695 to factor 2, r = 0.887 to total score and *P* < 0.001.

#### Internal consistency

The adapted questionnaire presented good internal consistency to factor 1 (α = 0.78, item range from 0.74 to 0.83) and moderate internal consistency to factor 2 (α = 0.56, item range from 0.45 to 0.65).

#### Internal structure: factor analysis

**Fig. 2:**
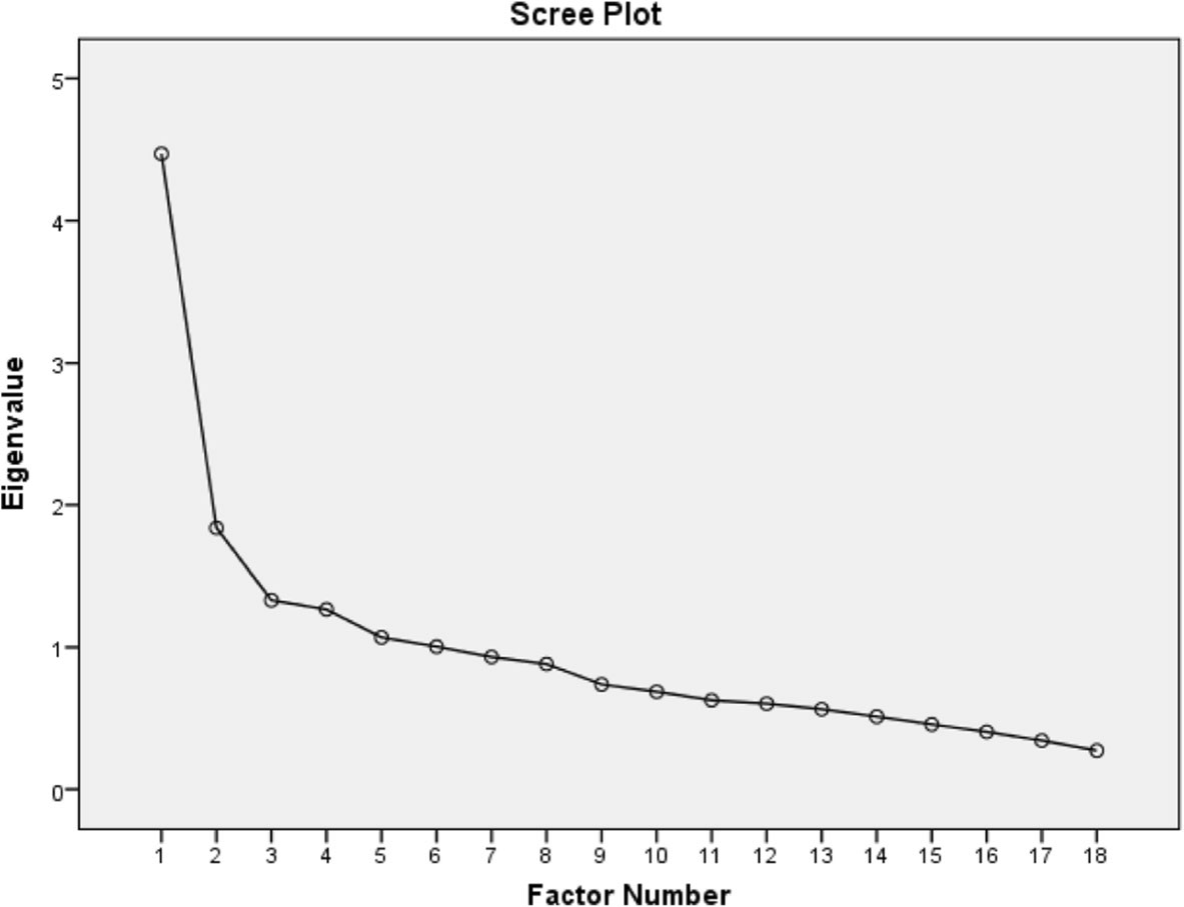
Scree plot of the exploratory two factor solution

The Kaiser-Meyer-Olkin values (0.773) and Bartlett’s Test of Sphericity (*P* < 0.001) indicated that the correlation matrix for the POS-S Renal was suitable for MLE. The factor analysis revealed that 35.05% of the variance could be explained with two factors with an eigenvalue higher than 1 and 10% of variance explained, and 61.01% with six factors with an eigenvalue higher than 1. The results are shown in Table 2. These six factors were used for the rotated component matrix. The scree plot indicated a two-factor solution (see Fig. 2). The item loading

**Table 2:** Total Variance Explained

Factor	Eigenvalues		
	Total	% of Variance	Cumulative %
1	4.472	24.843	24.843
2	1.838	10.210	35.053
3	1.332	7.398	42.450
4	1.267	7.037	49.488
5	1.070	5.943	55.431
6	1.005	5.582	61.013

**Fig. 3:**
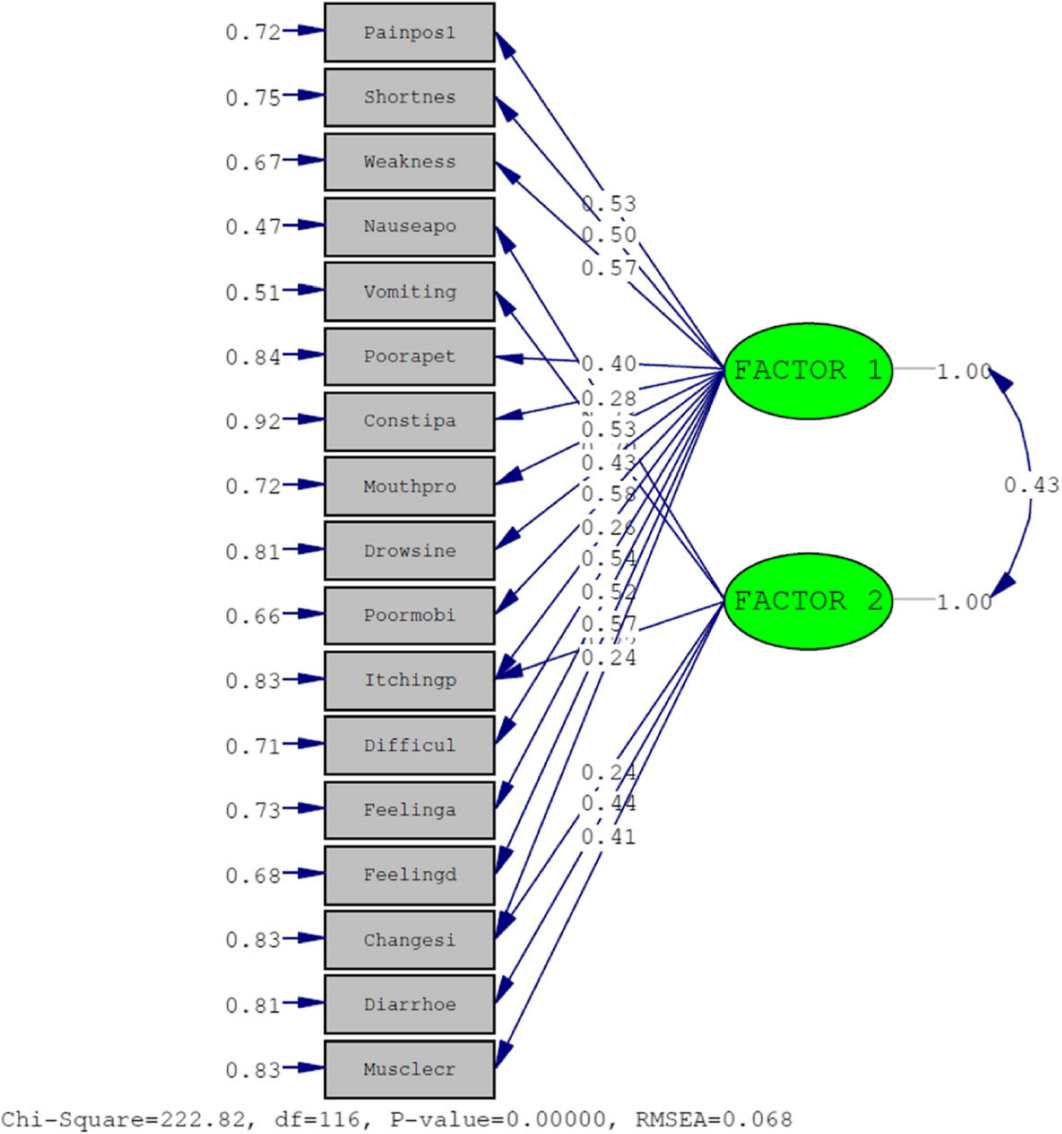
Confirmatory factor analysis of the two factor model of the Spanish version of POS-S Renal

Extraction Method: Maximum Likelihood

a. When factors are correlated, sums of squared loadings cannot be added to obtain a total variance

for the two-factor solution is shown in Table 3. The fit indices of the two factor model indicated an excellent fit: *χ* ^2^ = 222.82, RMESA = 0.68 and CFI = 0.98 (Fig. 3) [31].

**Table 3:** Factor Structure of Rotated Component Matrix

Item	Component	
	1	2
Pain	0.555	−0.162
Shortness of breath	0.497	−0.318
Weakness or lack of energy	0.547	−0.379
Nausea	0.251	−0.779
Vomiting	0.196	−0.694
Poor appetite	0.398	−0.236
Constipation	0.305	−0.001
Mouth problems	0.558	−0.305
Drowsiness	0.433	−0.320
Poor mobility	0.672	−0.050
Itching	0.361	−0.317
Difficulty sleeping	0.506	−0.386
Restless legs	0.150	−0.223
Feeling anxious	0.537	−0.303
Feeling depressed	0.586	−0.218
Changes in skin	0.329	−0.345
Diarrhoea	0.187	−0.486
Muscle cramps	0.229	−0.358

Extraction Method: Maximum Likelihood. Rotation Method: Oblimin with Kaiser Normalisation. Suppression at 0.35

#### Concurrent criterion-related validity

The correlation between the POS-S Renal and the MSAS-SF was confirmed. The POS-S Renal showed adequate correlations with the MSAS-SF. The values of the correlations were 0.809 (factor 1 – MSAS-SF total score), 0.518 (factor 2 – MSAS-SF total score) and 0.860 (POS-S Renal total score – MSAS-SF total score). The results demonstrated the value of the POS-S Renal as a multidimensional measure.

## Discussion

The translation and cross-cultural adaptation of the POS-S Renal into Spanish using recognised international guidelines was achieved satisfactorily. To our knowledge, this study is the first cross-cultural adaptation and psychometric analysis of the POS-S Renal. The study sample included dialysis and conservatively managed patients. Although the POS-S Renal is an instrument to measure symptoms in conservatively managed ACKD patients, in our experience it has been a useful clinical assessment tool for the dialysis population. We tested responses from patients in these two categories, and the results indicated that there were no significant differences between the two groups, although a trend towards a significant difference in the responses between the groups for factor 1 was found.

Excellent content validity of the Spanish modified version was demonstrated, which indicates that all items of this instrument are relevant for the measurement of the symptomatology in ACKD [23, 24]. This instrument showed satisfactory psychometric characteristics in terms of reliability [27], structural validity [31], and concurrent criterion-related validity [32]. The sample size was adequate for all analyses [33].

We added ‘cramps’ to the original 17-item POS-S Renal because of the frequency of cramps among patients with ACKD [4, 5]. Although in our study the prevalence of cramps was lower than has been found in other studies on the Spanish population, this is a common symptom that is often under-evaluated and is an important cause of the early termination of dialysis sessions [34, 35].

The test–retest reliability was high (r = 0.887 to total score), with values beyond those found in the modified ESAS in dialysis patients (r = 0.7 to total score) [11].

Internal consistency analysis indicated a satisfactory degree of interrelatedness among the items of the instrument [27]. Factor 1(α = 0.78) and factor 2 (α = 0.56) showed the highest and lowest results, respectively, with values below those found in the Arabic translation and modification of the DSI (Cronbach’s α = 0.91 overall) [36].

The two-factor solution obtained in the EFA accounted for a significant proportion of variance and showed support for the presence of construct validity, which provides support for evidence of the instrument validity. The factor analysis performed to evaluate the internal structure of the POS-S Renal confirmed the two-factor model, in line with other symptom assessment tools [25]. However, we performed a CFA, and the fit indices of the CFA model were satisfactory [29]. Nonetheless, two POS-S Renal items, namely, restless legs and changes in skin, did not load on either of these two factors. Consequently, it appears that these symptoms function as individual items. A possible explanation for this is that these two symptoms are best regarded as ‘causal indicators’ not ‘indicator variables’, and this suggests inherent problems with the application of the factor analysis [37]. Causal indicators are variables external to the patient that could affect symptoms, and indicator variables are unobservable variables which can be inferred from scores on multiple self-report items. Thus, the inclusion of indicator variables in factor analysis could lead to uninterpretable results [37, 38].

The concurrent criterion-related validity analysis with the MSAS-SF was supported by a strong correlation with factor 1 and total score, and a fair correlation with factor 2, which provides support for evidence that the scores of the POS-S Renal are an adequate reflection of a previously validated instrument that has been widely used in patients with ACKD. These results are in line with those found by the authors of the Arabic translation of the DSI and the modified ESAS with the Kidney Dialysis Quality of Life-Short Form (KDQOL-SF) [11, 36].

The extent and severity of the symptom burden in patients with ACKD demonstrates the importance of using an appropriate clinical tool [4, 5, 13, 15]. Likewise, the multidimensional nature of the symptoms should be considered in these patients [39]. The POS-S Renal is a validated instrument that provides information about a large number of physical and psychological symptoms. This also demonstrates the value of POS-S Renal as a useful and clinically appropriate symptom assessment tool to facilitate a multidimensional symptom evaluation in ACKD.

A patient with many mild symptoms can have a score identical to a patient with fewer, but more distressing symptoms, and a high score on even a single symptom could indicate a high level of patient distress for one symptom even if the patient’s total score is low. Thus, clinicians must look at the patient’s score on each of the 18 Spanish modified version of the POS-S Renal symptoms on its own, even though a total score can be obtained.

This study provides access to a PRO instrument to assess symptoms in CKD stage 4–5 for Spanish speaking populations. This single-page 18-item PRO is a self-administered questionnaire that is comprehensible and easy to complete. Hence, this study shows that this instrument will be of value in the symptom assessment of patients with ACKD in clinical practice and in research. Although this study contributes by filling the knowledge gap on the validation and usefulness of POS-S Renal, as well as, on symptoms assessment in ACKD, subsequent studies should be carried out to develop more refined measuring tools in this area [40].

The limitations to consider in this study include the lack of longitudinal data concerning other psychometric characteristics. Although our study shows a preliminary analysis of the psychometric properties of a modified POS-S Renal, sensitivity to change and minimally important differences were not evaluated. Further work using longitudinal data to determine responsiveness is needed to define the use of these measures in cohort studies and clinical trials that evaluate interventions. Finally, given the lower internal consistency for factor 2, a confirmatory factor analysis in a wide sample should be carried out to explore and interpret the behaviour of this factor.

## Conclusions

The Spanish modified version of the POS-S Renal demonstrated a two-factor structure and provided a PRO specific to the Spanish population that is valid and reliable. This clinical tool is simple to complete and easily understood. This instrument is comprehensive and can capture the multidimensional aspects of a range of symptoms. Consequently, the POS-S Renal can be recommended for clinical and research purposes in Spanish speaking populations.

## Additional files

Additional file 1: The questionnaire. It contains the Spanish modified version of the POS-S Renal that has been validated by the authors in this study. (DOCX 19 kb)
Additional file 2: Pilot study. It contains the results of the pilot study. (DOCX 46 kb)

## Abbreviations

ACKD: Advanced chronic kidney disease
ANOVA: Analysis of variance
CFA: Confirmatory factor analysis
CFI: Comparative fit index
CKD: Chronic kidney disease
CVI: Content validity index
DSI: Dialysis Symptom Index
EFA: Exploratory factor analysis
ESAS: Edmonton Symptom Assessment System
GDI: Global Distress Index
HRQoL: Health-related quality of life
ICC_2.1_: Intraclass correlation coefficient type 2.1
KDQOL-SF: Kidney Dialysis Quality of Life-Short Form
KS: Kolmogorov-Smirnov
MLE: Maximum likelihood extraction
MSAS-SF: Memorial Symptom Assessment Scale-Short Form
PHYS: Physical Symptom Distress Score
POS-S Renal: Palliative care Outcome Scale-Symptoms Renal
PRO: Patient reported outcome
PSYCH: Psychological Symptom Distress Score
RMSEA: Root mean square error of approximation
TNS: Total number of symptoms
*χ*^2^: Chi-square
