# Supplementary material for: Spanish modified version of the palliative care outcome scale–symptoms renal: cross-cultural adaptation and validation
Source: BMC Nephrol. 2016 Nov 18;17:180. doi: 10.1186/s12882-016-0402-8 (PMC5116210; doi:10.1186/s12882-016-0402-8)
Supplement: Additional file 1: — The questionnaire. It contains the Spanish modified version of the POS-S Renal that has been validated by the authors in this study. (DOCX 19 kb) [file 12882_2016_402_MOESM1_ESM.docx]

POS-S RENAL (versión española modificada) - **A CUMPLIMENTAR POR EL PACIENTE**

Debajo aparece una lista de síntomas que usted puede haber experimentado o no. Por favor, ponga una cruz en la casilla que corresponda para indicar cómo le han afectado cada uno de estos síntomas y cómo se ha sentido a lo largo de la semana pasada.

|  | **Nada**  Sin efecto | **Ligeramente**  pero no lo suficiente como para tratarlo | **Moderadamente**  limita algunas actividades o la concentración | **Fuerte**  las actividades o la concentración se ven afectadas notablemente | **Insoportable**  incapaz de pensar en otra cosa |  |
| --- | --- | --- | --- | --- | --- | --- |
| Dolor | □_0_ | □_1_ | □_2_ | □_3_ | □_4_ |  |
| Dificultad para respirar | □_0_ | □_1_ | □_2_ | □_3_ | □_4_ |  |
| Debilidad o falta de energía | □_0_ | □_1_ | □_2_ | □_3_ | □_4_ | **POS-S RENAL** |
| Náuseas (como si sintiera que va a vomitar) | □_0_ | □_1_ | □_2_ | □_3_ | □_4_ | **Palliative care Outcome Scale- Symptoms Renal** |
| Vómitos | □_0_ | □_1_ | □_2_ | □_3_ | □_4_ |  |
| Poco apetito | □_0_ | □_1_ | □_2_ | □_3_ | □_4_ |  |
| Estreñimiento | □_0_ | □_1_ | □_2_ | □_3_ | □_4_ |  |
| Problemas en la boca | □_0_ | □_1_ | □_2_ | □_3_ | □_4_ |  |
| Somnolencia | □_0_ | □_1_ | □_2_ | □_3_ | □_4_ |  |
| Poca movilidad | □_0_ | □_1_ | □_2_ | □_3_ | □_4_ |  |
| Picor | □_0_ | □_1_ | □_2_ | □_3_ | □_4_ |  |
| Problemas para dormir | □_0_ | □_1_ | □_2_ | □_3_ | □_4_ |  |
| Piernas inquietas o dificultad para mantener las piernas quietas | □_0_ | □_1_ | □_2_ | □_3_ | □_4_ |  |
| Sensación de angustia | □_0_ | □_1_ | □_2_ | □_3_ | □_4_ | **NOMBRE:** |
| Se siente deprimido | □_0_ | □_1_ | □_2_ | □_3_ | □_4_ |  |
| Cambios en la piel | □_0_ | □_1_ | □_2_ | □_3_ | □_4_ |  |
| Diarrea | □_0_ | □_1_ | □_2_ | □_3_ | □_4_ |  |
| Calambres | □_0_ | □_1_ | □_2_ | □_3_ | □_4_ | **NÚMERO DE PACIENTE:** |
| Cualquier otro síntoma: |  |  |  |  |  |  |
|  | □_0_ | □_1_ | □_2_ | □_3_ | □_4_ |  |
|  | □_0_ | □_1_ | □_2_ | □_3_ | □_4_ |  |

¿Qué síntoma le ha afectado más?_____________________________________________

¿Qué síntoma ha mejorado más?______________________________________________
