# Supplementary material for: Spanish modified version of the palliative care outcome scale–symptoms renal: cross-cultural adaptation and validation
Source: BMC Nephrol. 2016 Nov 18;17:180. doi: 10.1186/s12882-016-0402-8 (PMC5116210; doi:10.1186/s12882-016-0402-8)
Supplement: Additional file 2: — Pilot study. It contains the results of the pilot study. (DOCX 46 kb) [file 12882_2016_402_MOESM2_ESM.docx]

**Results**

**Pilot study**

The pilot study involved 30 ACKD patients. There was a low proportion of missing responses (this did not exceed 2%). The descriptive characteristics of the subjects who agreed to complete the questionnaires are shown in Table 1.

**Table 1. Sociodemographic and Clinical Characteristics of the Sample (N=30)**

**Characteristics Total**

**(N=30)**

Age (mean, SD) 71.6 (±16)

Gender

Male 17 (56.7%)

Female 13 (43.3%)

Ethnicity

Caucasian 30 (100%)

Spanish descent 30 (100%)

Marital status

Married 17 (56.7%)

Not married 13 (43.3%)

Causes of CKD

Renal vascular disease 15 (50%)

Diabetic nephropathy 10 (33.3%)

Primary glomerular disease 1 (3.3%)

Unknown aetiology 4 (13.3%)

Barthel index (mean, SD) 80 (±23)

Charlson comorbidity index 5.3 (±2.3)

***Reliability and internal consistency***

The values obtained after the study on the reliability by calculating the ICC (2, 1), and the corresponding Cronbach α are shown in Table 2.

**Table 2. Reliability and internal consistency**

**Item Cronbach Lower Bound Upper Bound**

**alpha ICC (2,1) 95%CI ICC (2,1) 95%CI**

Pain 0.990 0.957 0.990

Shortness of breath 0.971 0.940 0.986

Weakness or lack of energy 0.968 0.934 0.985

Nausea 0.695 0.359 0.855

Vomiting 0.653 0.270 0.835

Poor appetite 0.965 0.927 0.984

Constipation 0.960 0.917 0.981

Mouth problems 0.979 0.956 0.990

Drowsiness 0.974 0.944 0.987

Poor mobility 0.988 0.975 0.994

Itching 0.987 0.973 0.994

Difficulty sleeping 0.984 0.967 0.993

Restless legs 1.000 1.000 1.000

Feeling anxious 0.980 0.957 0.990

Feeling depressed 0.978 0.954 0.990

Changes in skin 0.953 0.901 0.978

Diarrhoea 0.494 -0.62 0.759

Muscle cramps 0.959 0.914 0.980

Total 0.984 0.966 0.992

***Internal structure: factor analysis***

Kaiser-Meyer-Olkin measure of sampling adequacy had an score of 0.545, and Bartlett’s test of

sphericity was significant (P < 0.001). The results obtained in the calculation of the internal structure through exploratory factor analysis (EFA) with maximum likelihood extraction (MLE), where 67.875% of the variance could be explained with five components with an eigenvalue higher than 1, are shown in Table 3. The scree plot is also shown in Fig 1.

**Table 3. Total variance explained**

| **Factor Eigenvalues**  **Total % of Variance Cumulative %** |
| --- |
| 1 4.818 28.343 28.343  2 2.323 13.662 42.005  3 1.905 11.203 53.209  4 1.365 8.028 61.236  5 1.129 6.639 67.875  6 0.970 5.707 73.582  Extraction Method: Maximum Likelihood  **Figure 1. Scree plot** |


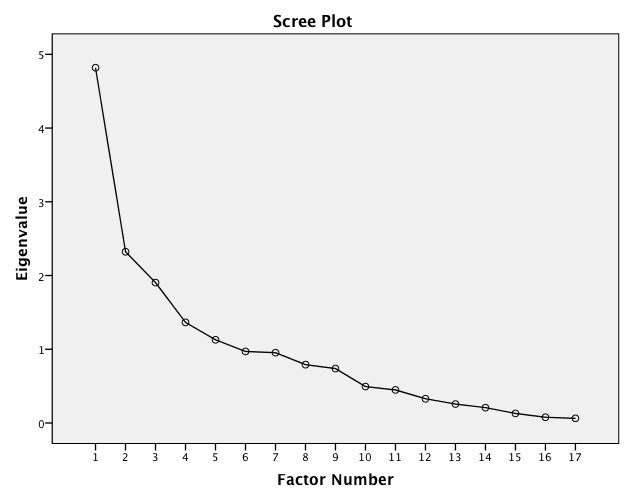


***Concurrent criterion-related validity***

With regard to convergent validity, the a priori hypothesis of the correlation between the POS-S Renal and the MSAS-SF was confirmed. The POS-S Renal showed adequate correlations with the MSAS-SF. The values of the correlations were 0.938 (POS-S Renal total score – MSAS-SF total score).

|  |
| --- |
